# The Bradyzoite: A Key Developmental Stage for the Persistence and Pathogenesis of Toxoplasmosis

**DOI:** 10.3390/pathogens9030234

**Published:** 2020-03-21

**Authors:** Aude Cerutti, Nicolas Blanchard, Sébastien Besteiro

**Affiliations:** 1 LPHI, UMR 5235, CNRS, University of Montpellier, 34095 Montpellier, France; 2 Center for Pathophysiology Toulouse-Purpan (CPTP), INSERM, CNRS, University of Toulouse, 31024 Toulouse, France

**Keywords:** *Toxoplasma gondii*, chronic toxoplasmosis, persistence, latency, bradyzoite, differentiation, metabolism

## Abstract

*Toxoplasma gondii* is a ubiquitous parasitic protist found in a wide variety of hosts, including a large proportion of the human population. Beyond an acute phase which is generally self-limited in immunocompetent individuals, the ability of the parasite to persist as a dormant stage, called bradyzoite, is an important aspect of toxoplasmosis. Not only is this stage not eliminated by current treatments, but it can also reactivate in immunocompromised hosts, leading to a potentially fatal outcome. Yet, despite its critical role in the pathology, the bradyzoite stage is relatively understudied. One main explanation is that it is a considerably challenging model, which essentially has to be derived from in vivo sources. However, recent progress on genetic manipulation and in vitro differentiation models now offers interesting perspectives for tackling key biological questions related to this particularly important developmental stage.

## 1. Introduction

**Figure 1:**
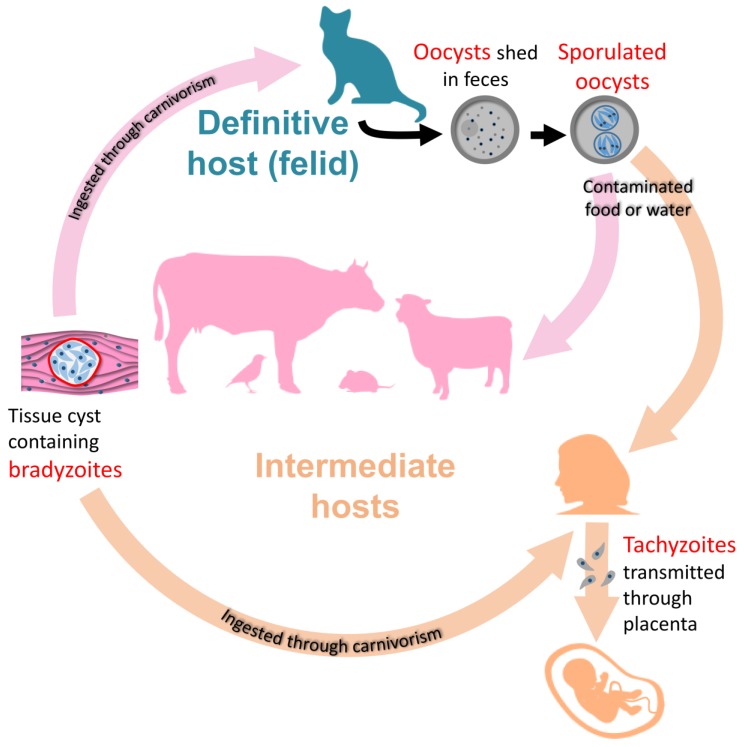
The life cycle of *Toxoplasma gondii*. Bradyzoites occupy a central place in the transmission of the parasite, between the intermediate, or to the definitive hosts.

*Toxoplasma gondii* is a parasite whose life cycle classically includes transmission from definitive hosts (felids), in which sexual reproduction occurs, to intermediate hosts (warm-blooded vertebrates), in which it multiplies asexually [1] (Figure 1). This parasite has an unparalleled range of intermediate hosts: it is believed to infect up to a third of the world’s human population as well as a broad variety of animal species. 

Transmission from definitive hosts to intermediate hosts is mediated by oocysts shed by the former in the environment. These robust parasite stages may survive for several years and can infect intermediate hosts through water and food contamination [2]. Upon infection, the parasites differentiate into an asexually-dividing form called tachyzoite, whose rapid replication and spread within the body of the intermediate host may cause a disease called toxoplasmosis [3]. This infection phase is usually contained by the immune system in immunocompetent individuals. However, immunocompromised individuals are at risk of developing serious, and possibly fatal, complications such as encephalitis. Besides, vertical transmission to the fetus from an acutely-infected mother is also an important cause of the severe and disabling disease [4] (Figure 1).

In healthy and immunocompetent individuals, tachyzoites are not completely eliminated by the immune system, but they differentiate into slowly-growing bradyzoite forms, establishing within tissue cysts, primarily in the central nervous system and muscle [5]. This persistent chronic form of the pathogen may remain for a long time in the host (perhaps throughout its life [6]), which is a major concern as it may reactivate and lead to a severe pathology in the event of a weakened immune system. Because of their ability to persist for a long time in the intermediate hosts, bradyzoites play a central role in the life cycle of the parasite (Figure 1). The classical completion of the cycle includes predation of an intermediate host by felids, and thus ingestion of parasite-containing tissue cysts. They then differentiate into sexually-competent stages that lead to the formation of the oocysts that are shed by the felid in the environment to start another cycle [1].

The ability for the parasites to persist as bradyzoite-containing cysts for a long time in the intermediate host certainly improves the opportunities for transmission to a definitive host by predation, and thus completion of the cycle. Besides, in addition to the prey/predator cycle that is futile in the case of a large intermediate host felids cannot prey on, the parasites can still be transmitted in the form of tissue cysts to other intermediate hosts by carnivorism (Figure 1). *T. gondii* can thus proliferate as asexually-dividing forms, and persistent bradyzoite stages are clearly important to ensure transmission to a new host, be it intermediate or definitive. 

Although they clearly have a key role in transmission and pathogenesis, bradyzoites have been largely overlooked as study models. This is most likely due to the fact that they are not easily manipulated in vitro, and that in vivo experiments do not allow a straightforward investigation of key aspects of bradyzoites biology such as their metabolic requirements or their interaction with the host. In this review, we summarize important aspects of their differentiation and persistence mechanisms, as well as recent technical progress that will hopefully enable tackling some important biological questions related to this developmental stage in the near future.

## 2. Importance of the Bradyzoites for the Pathology of Toxoplasmosis

**Figure 2:**
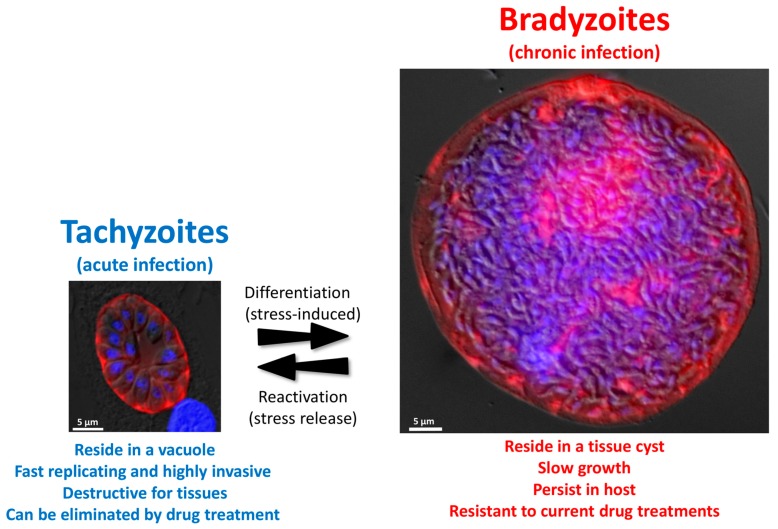
*T. gondii* can be found in intermediate hosts as two different stages called tachyzoite and bradyzoite. Images represent an in vitro grown tachyzoite-containing vacuole (left) and a bradyzoite-containing tissue cyst extracted from a mouse brain (right). The parasitophorous vacuole membrane was labelled with an anti-GRA3 antibody (red, left), the cyst wall was labelled with *Dolichos biflorus* agglutinin (red, right). DNA was labelled with 4′,6-diamidino-2-phenylindole (DAPI, blue). Images represent merged fluorescence and differential interference contrast (DIC) micrographs.

The outcome of infection with *T. gondii* is dependent on the interplay between host and parasite factors. Tachyzoites are actively invading host cells [7]. Through this process, they establish themselves within a parasitophorous vacuole (PV) in which they multiply (Figure 2), and then actively egress to invade neighboring cells. This so called lytic cycle [8], when repeated multiple times, will cause considerable tissue damage and is responsible for the symptoms of the acute phase of the disease. Tachyzoites will also spread rapidly from the initial site of infection (the intestine) to distant tissues via the blood flow and the lymphatic system [9,10].

### 2.1. Escaping the Immune System

Generally, upon primary infection, the host will develop a number of anti-parasitic mechanisms that first include innate immunity, but also adaptive and cell-autonomous responses [11]. The parasite has also evolved strategies to successfully bypass or manipulate the immune system, noticeably by secreting proteins that modify host transcriptional programs or signaling pathways [12,13]. Thus, some tachyzoites are not completely cleared out by the host immune response, and they manage to convert to the bradyzoite stage [14,15]. This resistant form, thanks to a very slow replication, a reduced metabolic activity, and its confinement within a cyst, manages to escape elimination by the immune system and ensures persistence of the parasite. The establishment of chronic infection thus involves a fine balance between host immunity and parasite evasion of this immune response.

### 2.2. The Differentiation Process 

Differentiation of tachyzoites into bradyzoites is likely triggered by stress conditions in vivo (Figure 2). It can also be induced in vitro by a number of methods, which include alkaline pH stress, heat shock, nutrient starvation, and the use of specific drugs (Table 1) [16,17,18,19,20,21,22,23,24,25,26,27,28,29]. This is a complex process that consists of extensive changes in parasite gene and protein expression. As intact cysts can be purified from brain tissue by isopycnic centrifugation, over the years, they have been used to raise several monoclonal antibodies, which provided markers to follow the kinetics of conversion [30,31]. Using these antibodies or fluorescent reporter proteins, it was shown by microscopic observation that the in vitro differentiation process happens over the course of several days [32,33]. Before reaching a fully mature bradyzoite stage, the conversion occurs as a continuum through transitional intermediate stages co-expressing tachyzoite and bradyzoite proteins. Importantly, the dynamic maturation of cysts has also been shown to occur in vivo, with some heterogeneity in parasite growth within the same cyst [34]. Strikingly, these observations highlighted that, although bradyzoites had long been considered to be dormant stages, even mature tissue cysts display some heterogeneity in replication patterns, hinting that low level of sporadic replication still happens within terminally-differentiated tissue cysts [34,35].

There are major changes accompanying differentiation into bradyzoites (Figure 2). Noticeably, the PV in which tachyzoites replicate is modified to become a heavily-glycosylated cyst wall that is several hundred nanometers thick [36,37], and contains many stage-specific proteins of yet unknown function [38,39]. In addition, several aspects of parasite metabolism change drastically. Metabolic enzymes can be expressed at different levels in tachyzoites and bradyzoites, or these may even express different stage-specific isoforms [40,41,42,43,44]. For instance, bradyzoites appear to rely on anaerobic glycolysis instead of aerobic respiration for energy production [45,46]. Strikingly, one ultrastructural characteristic of bradyzoites is that they accumulate cytoplasmic starch granules [47]. Although their function is not completely elucidated yet, these granules are hypothesized to serve as a long-term energy store during chronic infection, which could help maintaining parasite viability in low-nutrient niches, or serve as a rapidly available energy source for reactivation when they encounter favorable conditions [48,49].

The transition between tachyzoites and bradyzoites involves considerable changes in gene expression. Transcriptomic analyses of in vivo cysts obtained three or four weeks post-infection have revealed that the expression levels of hundreds had changed [50,51], while transcription profiles remained essentially stable three months post-infection [52]. How this is regulated is still largely a mystery. The control of gene expression is exerted by mechanisms involving complex interactions of transcriptional complexes with not only the DNA sequence itself, but also chromatin proteins [53]. In line with this, epigenetic modifications of histones have been shown to play a part in regulating the tachyzoite/bradyzoite interconversion [23,24,54,55]. An original feature of *T. gondii* is that it lacks many of the recognizable specific transcription factors operating in other eukaryotes, but instead expresses several members of the plant-like ApiAP2 DNA-binding family [56]. There is evidence that several ApiAP2 factors are involved in regulating bradyzoite development, with probably a complex interplay between tachyzoite-specific and bradyzoite-specific ApiAP2 factors repressing or activating differentiation, respectively [57,58,59,60]. However, none of these factors was identified as the sole responsible for controlling differentiation. Interestingly though, a Cas9-mediated genetic screening allowed the discovery of a transcription factor called BFD1, which seems to act as a master switch for differentiation into bradyzoites, as it is both necessary and sufficient for this process [61]. Because this factor is unrelated to the ApiAP2 family, it also highlights an unsuspected large diversity and complexity in the control of the differentiation process.

### 2.3. Reactivation

Importantly, the differentiation process is reversible (Figure 2). Upon stress removal in vitro, or when tissue cyst bradyzoites (which are fully competent for host invasion) are used to infect host cells in permissive conditions, they can differentiate back into tachyzoites in a matter of a few days [32]. Again, not much is known about how the differentiation of bradyzoites back to the tachyzoite form is regulated at the transcriptional level, although there is likely a balance of expression between stage-specific transcription factors [58]. In vivo, this has serious implications for the pathology. Tissue cysts may occasionally rupture [62] and released parasites are probably cleared by the immune system in immunocompetent hosts. Interferon gamma (IFNγ) and its production by CD8^+^ T-cells are essential for controlling the proliferation of *T. gondii* tachyzoites [63,64]. For instance, anti-IFNγ treatment of chronically-infected mice results in reactivation of encysted parasites [65]. In a pathological context, bradyzoite reactivation can cause encephalitis which may lead to death in immunodeficient patients, including organ transplant recipients [66] or HIV patients [67]. In summary, tissue cysts likely do not cause major pathological damage, as parasites which may be eventually released as a result of stochastic bradyzoite reactivation are usually promptly eliminated by the immune system. However, in some conditions, cyst rupture can lead to a pathogenic mechanism of toxoplasmic encephalitis [68]. A watchful immune system is thus key for the control of parasite interconversion and thus for the balance between acute and chronic toxoplasmosis.

## 3. Immune Detection and Control of Bradyzoites

With the exception of fully refractory mouse or rat strains, it is assumed, based on several experimental animal models, that primary exposure to *T. gondii* elicits a detectable adaptive immune response (e.g., positive serology), and that the host is unable to mount sterilizing immunity. Host response modulation by tachyzoites undoubtedly provides the parasite with a selective advantage during the acute phase, allowing it to set in place bradyzoite differentiation programs in the reservoir tissues. In addition, some evidence, in particular related to antigen presentation as described below, suggest that bradyzoites are more ‘immunologically silent’ than tachyzoites, thereby adding another strategy for the establishment of a persistent infection.

This notion of immune evasion applies to the major histocompatibility complex (MHC) I-dependent CD8^+^ T-cell recognition of parasite-infected cells in the central nervous system (CNS). Although several cell types can be infected by *T. gondii* in the CNS, a conditional MHC I invalidation model showed that parasite control in vivo strongly relies on MHC I presentation of *T. gondii* antigens by infected neurons [69], which are the main cell type supporting bradyzoite development in the CNS [70]. Moreover, efficient parasite control occurs regardless of whether the protective antigen is expressed in bradyzoites or not, indicating that CD8^+^ T-cell surveillance is mostly achieved on tachyzoite-infected cells [69]. These results are consistent with earlier 2-photon microscopy studies showing that parasite-specific CD8^+^ T-cells interact predominantly with tachyzoite-infected and non-infected antigen-presenting cells, while they largely ignore cysts [71,72]. Up to now, the reasons for such an immune evasion are unclear. One hypothesis is that the cyst wall may limit the passage of antigens in the neuronal cytosol and hinder their subsequent MHC I presentation. Two exported parasite effectors, GRA16 and GRA24, indeed appear to be blocked within the lumen of in vitro-differentiated cysts [73]. However, several *T. gondii* proteins have been found to be efficiently sorted to the cyst membrane [39], including the protective GRA6 antigen [39,69], whose presence at the PVM is known to favor its MHC I presentation [74]. Also arguing against a limitation of the antigen efflux, the permeability of the cyst wall for small molecules appears to be even higher than that of the PV limiting membrane (~10 kDa or less, versus ~2 kDa or less, respectively [37,75]). Alternatively, it is possible that, through the export of effectors beyond the cyst wall, bradyzoites actively suppress MHC I presentation in *cis* or in *trans* in the chronically-infected CNS. However, to this day, it remains unclear which, if any, of the already described exported effectors are relevant in neuronal cysts. Future discovery of dual-stage or bradyzoite-specific effectors that may hijack neuron and muscle cells should help elucidate this puzzling question.

In addition, while there is no doubt that immune responses fall short of clearing chronic *T. gondii* stages in reservoir tissues, it does not necessarily mean that immune detection of bradyzoites does not occur at all. Several lines of evidence support the idea that bradyzoites are not completely ‘immunologically silent’. First, the chitin-rich cyst wall is detected by alternatively activated macrophages recruited to the CNS, which in turn produce chitinase and are able to disrupt the cysts [76]. Second, humoral responses observed in chronically-infected hosts do target bradyzoite-specific proteins (e.g., MAG1 [77]), suggesting that germinal center reactions involving bradyzoite antigen-specific B-cells and follicular helper CD4^+^ T-cells can take place.

In conclusion, while immune control is clearly not as effective on bradyzoites as it is on tachyzoites, the exact contribution of bradyzoite-specific modulatory effectors in the failure of the host immune system to eliminate the chronic stages remains poorly understood.

## 4. Current Research Strategies for Interfering with the Tachyzoite-Bradyzoite Interconversion

The flexibility of stage interconversion and the long term persistence of *T. gondii* as bradyzoite-containing tissue cysts constitute a durable threat to intermediate hosts. Besides, although cyst-enclosed bradyzoites are supposedly harmless to immunocompetent individuals, several studies suggested the persistence of *T. gondii* in the brain may be linked to neurological diseases and neuropsychiatric disorders such as schizophrenia, epilepsy, Parkinson’s disease, and cancer [78,79,80,81,82,83]. Importantly, the general consensus is that tissue cysts are resistant to drugs commonly used to treat acute toxoplasmosis, such as atovaquone, pyrimethamine, and sulfadiazine [84,85,86]. Consequently, there is no currently approved therapy able to cure chronic infection, and strategies that would lead to a complete eradication of encysted parasites are thus needed. Drugs targeting both acute and chronic toxoplasmosis would be particularly useful. In that regard, it is for instance quite interesting that small inhibitors of CDPK1 (a Ca^2+^-dependent protein kinase regulating parasite motility, cell invasion, and egress) can treat acute toxoplasmosis, but also prevent bradyzoite reactivation [87]. In addition, as described in this section, recent advances on fundamental aspects of bradyzoite biology now offer potentially interesting perspectives for preventing cyst formation and persistence, or impacting bradyzoite viability.

### 4.1. Blocking the Differentiation Process by Shutting down Transcriptional or Translational Programs

Strategies that aim at blocking the differentiation process by turning down transcription of bradyzoite-specific genes and/or translation of bradyzoite-specific proteins may prevent the generation of encysted parasites, which would have no alternative but to remain as tachyzoites. It may also be possible to modulate transcriptional programs in order to force bradyzoites to differentiate into tachyzoites. Then, remaining tachyzoites would likely be cleared by the immune system in immunocompetent individuals. Of course, these may be risky therapeutic strategies in the context of immunocompromised individuals, so drugs that effectively eliminate tachyzoites should be co-administered.

Transcription factors may be targeted by preventing their binding to DNA, modulating their expression or degradation, and blocking protein/protein interactions. Altering the epigenetic control of bradyzoite gene expression by modulating the acetylation of specific histones is also something that may be envisaged [23,24,55]. Several ApiAP2 factors have been involved in the transcriptional program that is specifically triggered during differentiation, however, inactivation of individual genes did not allow complete ablation of the differentiation process. It should however be noted that depletion of the AP2IV-4 factor causes mis-timing of bradyzoite protein expression which, even if it does not prevent formation of cysts in vitro, clearly affects the establishment of chronic disease in mice by eliciting a more efficient immune response [60]. The recently-characterized BFD1 transcription factor seems on the contrary to be acting as a main regulator of differentiation, as gene knockout leads to a complete inability to generate cysts [61]. In addition, *ΔBFD1* parasites may have a potential as an attenuated strain to be used as a prophylactic vaccine, because they may proliferate as tachyzoites and elicit an immune response but would be unable to durably establish themselves as a resistant latent form. Again, with the caution that only fully immunocompetent individuals should be treated with such a vaccination strategy.

Conversion into latency is triggered by environmental stresses. Eukaryotic cells have a conserved adaptive integrated stress response pathway, in which phosphorylation of the alpha subunit of the translation initiation factor eIF2 (eIF2α) by stress-sensing kinases leads to a decrease in global protein synthesis and, on the other hand, to the induction of selected genes involved in the stress response [88]. Accordingly, in *T. gondii*, translational control seems to help maintaining latency [89] and eIF2α remains highly phosphorylated in bradyzoites [90]. Guanabenz, a selective eIF2 phosphatase inhibitor has been shown to increase *T. gondii* eIF2α phosphorylation, to induce bradyzoite gene expression and the conversion of tachyzoites into bradyzoites, and to impede the reconversion of bradyzoites into tachyzoites in vitro [90,91,92]. However, upon treatment with guanabenz, a significant proportion of the bradyzoite-containing cysts appeared to have an abnormal morphology [92]. Most interestingly, in vivo experiments have shown that this drug can in fact reduce the cyst burden in the brains of chronically-infected mice [92], and it also suppresses parasite-induced behavioral changes in infected animals [93]. In conclusion, translational control mechanisms during encystation seem to be providing potentially interesting new drug targets.

### 4.2. Interfering with the Biogenesis or the Integrity of the Cyst Wall

During the course of chronic infection, there is a progressive transformation of the PV into a cyst wall. This structure is organized into distinct layers: a compact outer layer beneath the limiting cyst membrane, and a less densely compacted inner layer that faces the cyst matrix. The mechanisms for building a cyst wall compartment beneath the limiting cyst membrane during differentiation still need to be determined, but a number of proteins secreted from compartments called dense granules seem to play an important role [94]. The cyst wall material is heavily glycosylated, and glycoproteins such as PPG1 [95] or CST1 [96] are important for the formation of the cyst wall structure. Of note, CST1 is the selective binding target of the *Dolichos biflorus* agglutinin (DBA), which has high affinity for the glycosylated wall structure and is commonly used to detect cysts (Figure 2). 

Large in vivo cysts can contain hundreds of parasites and are usually in the range of 25 to 70 µm in size (Figure 2) [34,36,37]. There are complex invaginations of the limiting membrane of the cyst wall that may represent a way of increasing the surface area and thus would facilitate exchange of material between the cyst matrix and the host cell. The vesicles, elongated tubules between bradyzoites, and filamentous material observed in the cyst matrix may thus reflect a complex trafficking network that could be used for nutrient acquisition [37]. As the cyst wall seems to be permeable to molecules which are 10 kDa or less [37], it is likely parasites within the cysts have access to nutrients from the cytoplasm of the host [97]. Finally, as mentioned in part 3, the latent tissue cysts are supposed to largely evade the immune response, although the part played by the cyst wall in limiting the interaction of the bradyzoites with the components of the immune response is not yet elucidated.

Given the potentially important roles for the cyst wall in the survival of bradyzoites, as a therapeutic approach it may be interesting to interfere with its biogenesis or its integrity. Unfortunately, very little is known about the composition of the cyst wall, or how it is built or derived from the PV membrane (PVM), but some progress has been made recently. An approach using transcriptomic analysis of differentially-expressed genes and selecting candidates with a putative signal peptide, allowed the identification of a few bradyzoite-secreted effectors as components of the cyst wall [50]. More recently, proteomics-based studies using either differential centrifugation and immuno-affinity isolation of cyst wall components, or proximity-based labeling, identified several new components of this structure and confirmed a number of other previously-characterized ones [38,39]. In the future, there is no doubt similar strategies will help identify important actors of cyst wall biology. As for now, the importance of glycoproteins for cyst wall integrity suggests enzymes or transporters involved in the glycosylation of cyst wall proteins, including *T. gondii* nucleotide-sugar transporter NTS1 [98] or the galactosaminyltransferase ppGalNAc-Ts [99], have potential as therapeutic targets to interfere with bradyzoite persistence.

### 4.3. Identifying Metabolic Weak Spots

A slow-down of parasite growth is preceding the induction of the bradyzoite differentiation program [100]. However, cyst-contained bradyzoites, instead of being completely blocked in growth as it was presumed for a long time, are now simply assumed to grow much slower and in a much more heterogeneous way than tachyzoites [34,35,101]. Bradyzoites thus probably also have metabolic needs which are non-negligible, although experimental evidence suggests some marked differences with tachyzoites. 

Various interactions with the host cell seem to have important implications for differentiation into bradyzoites and for their subsequent survival. Like for tachyzoites (albeit to a lesser extent), it has been recently shown that in vitro-generated cysts can recruit some host cell organelles, presumably as a nutrient source [102]. In vivo, tissue cysts are predominantly found in long-lived cells such as mature skeletal muscle and brain neurons [5]. Strikingly, tachyzoites have been shown to spontaneously convert to bradyzoites in vitro within neuronal and muscle cell types, which suggests a mechanism for sensing the cell division or the metabolic states of the host is important to induce bradyzoite development in physiological conditions ([103,104,105], more on this in part 5 of this review). Further investigations confirmed the cell division [29,106] and the metabolic [107] states of the host cells indeed play a role in the switch from the tachyzoite to the bradyzoite form. Inhibitors of mitochondrial functions promote the differentiation into bradyzoites, suggesting that alterations in the parasite metabolism are correlated to stage conversion [16,17].

Conversely, for energy generation anaerobic glycolysis seems to be favored in bradyzoites over mitochondrion-housed tricarboxylic acid cycle and oxidative phosphorylation [45,46]. In accordance with that, bradyzoites express stage-specific isoforms of glycolytic enzymes [40,41,42,44]. So far, the flexibility and redundancy of the pathways for generating energy remains largely unexplored in bradyzoites [108]. A very striking feature of these stages is the high numbers of starch granules made of amylopectin (a storage polysaccharide) [47], whose role is not completely elucidated, but which are likely used as an energy source by the parasite. The balance between degradation and storage of amylopectin appear to be finely regulated as both an accumulation or a lack of these granules seem detrimental for cyst development [49]. Consequently, alteration of the balance between amylopectin synthesis and digestion by interfering with the function of regulating factors such as the Ca^2+^-dependent protein kinase CDPK2 [48] or the enzyme glycogen phosphorylase [49], may be an interesting therapeutic option for the treatment of chronic toxoplasmosis.

Among the other important cellular building blocks, bradyzoites seem also to be able to acquire lipids from the host cell and to store them as lipid droplets [109]. A specific pharmacological inhibitor of the activity of acyl-CoA:diacylglycerolacyltransferase (DGAT), a key enzyme for triglyceride biosynthesis and lipid storage, impacts bradyzoite development, leading to parasite malformations within the cysts [109]. Like tachyzoites, bradyzoites are presumably auxotrophic for several amino acids [110], and thus depend on exogenous sources. The recycling of proteins and organelles is also an important source of metabolites for eukaryotic cells. This is usually mediated by the autophagy pathway, a self-digestive process whereby cellular material is sequestered in vesicles called autophagosomes and digested through the lysosomal compartment for subsequent recycling [111]. Interestingly, interfering with the proteolytic capacity of bradyzoites leads to an accumulation of autophagosomes and a markedly reduced chronic infection [112,113]. This suggests recycling by proteolysis, potentially through autophagy, is important for bradyzoite development. Whether it is to provide nutrients for the bradyzoites or for cellular remodeling during differentiation is, however, currently unknown. 

Besides the mitochondrion, *T. gondii* and several related apicomplexan parasites harbor another organelle of endosymbiotic origin called the apicoplast [114]. This non-photosynthetic plastid is also a major metabolic hub as it contains biosynthetic pathways for synthesizing fatty acids, isoprenoids, heme, and iron/sulfur clusters. It is key to the survival of tachyzoites, but its contribution to the viability of bradyzoites remains largely unexplored. Clearly, a better understanding of the metabolic capacity of bradyzoites and the contribution of specific organelles such as the mitochondrion or the apicoplast might provide important avenues for intervention toward the elimination of these persistent forms. However, there are several potential hurdles that should be taken in consideration when targeting metabolic pathways in bradyzoites. First, there is likely some heterogeneity in metabolic requirements within a single cyst. Depending on the parasites location within a large tissue cyst, they may be exposed to very variable conditions regarding oxygen or nutrient availability for example. In addition, division within a tissue cyst is asynchronous [33,34], thus metabolic requirements are probably variable depending on the parasites. Importantly, while tachyzoites remain connected and thus can potentially exchange metabolites during division, there is no obvious connection between parasites in cyst-contained bradyzoites [115]. While on the one hand this may render parasites more vulnerable to metabolism alteration, on the other hand, reduced or halted growth in part of the cyst bradyzoite population would presumably contribute to resistance to drugs targeting metabolism, which are more likely to impact actively-dividing parasites.

## 5. New Tools for Studying Bradyzoite Biology

Improving our understanding of bradyzoite biology is critical for the development of knowledge-based therapeutic strategies to eliminate latent infection. Recent advances in molecular engineering techniques and implementation of alternative cell biology models for in vitro differentiation provide interesting perspectives.

### 5.1. Improved in vitro Differentiation Models

For years, the bradyzoite stage was only investigated through parasite inoculations to rodents. However, not only large amounts of tissue cysts may be difficult to obtain without having to use many animals, but there is also a limited ability to control and modulate multiple experimental parameters in these animal models. Besides, developing alternative systems to animal models such as in vitro host cell models is becoming a major issue in biology, aiming to both reduce costs and overcome ethical limitations. Later, in vitro differentiation systems were developed, accelerating the discovery of the molecular mechanisms involved into *T. gondii* conversion from one stage to another. During the 1990s, in vitro models using mostly fibroblasts or macrophages as host cells, coupled with different types of stresses were established to facilitate cysts generation [16,20,21,25]. Since then, in vitro bradyzoite differentiation by alkaline stress has become the most commonly used strategy [116], although other kinds of stresses can induce a transition from tachyzoite to bradyzoite in different host cell models (Table 1). These include the use of heat shock treatment [21], IFNγ and pro-inflammatory cytokines or agents [20], different types of drugs and chemicals (such as histone deacetylase inhibitors [19,23,24], or mitochondrial inhibitors [16,17]), or nutrient starvation [18,22,26]. However, *T. gondii* stage conversion in vitro is an asynchronous and rather heterogeneous process, often leading to mixed parasite populations composed of both tachyzoites and bradyzoites. Moreover, a recent single cell transcriptomic analysis suggests alkaline stress-induced bradyzoites may be more heterogeneous than previously thought [101]. Besides, tissue culture conditions for bradyzoite development do not entirely model animal infections, so it is reasonable to wonder if in vitro-generated bradyzoites are really equivalent to bradyzoites harvested from tissue cysts. For instance, transcriptomic data revealed differences in expression between in vitro generated cysts and tissue cysts from mice brain [50,51,117]. 

**Figure 3:**
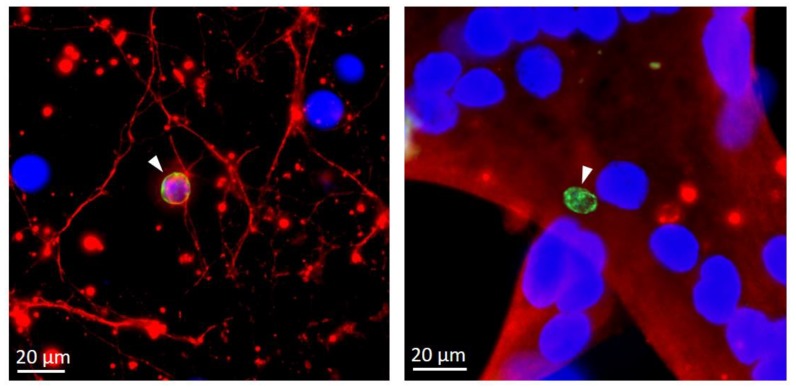
Spontaneous differentiation of bradyzoites in neurons and myotubes. Cysts (arrowhead, labelled with *Dolichos biflorus* agglutinin, green) form spontaneously in primary hippocampal neurons from mouse (left, labelled with anti- Microtubule Associated Protein 2, red) or immortalized human myotubes (right, labelled with anti-myosin heavy chain, red). DNA was labelled with with 4′,6-diamidino-2-phenylindole (DAPI, blue).

It is also well known that the type [118,119] and the physiological state [29,106,107] of the host cells are critical determinants of stage conversion. In vivo, although cysts may be found in a variety of tissues during early stages of chronic infection [120], they persist essentially in post-mitotic neuronal and muscular cells [5], which may provide a suitable cellular environment for triggering bradyzoite formation or favor parasite persistence by a less rapid turnover. Interestingly, spontaneous and efficient differentiation in vitro is observed in these same cell types (Figure 3) and the last 20 years have seen the emergence of new in vitro host cell models taking advantage of this. The use of cell lines or primary skeletal muscle cells that can be differentiated into mature myotubes in vitro has been quite well documented [104,105,106,121,122]. Similarly, for neuronal models, several studies described the use of cell lines derived from neuronal tumors or primary neuronal cultures [103,123]. Recent developments of neurons derived from induced pluripotent stem cells, or through cellular reprogramming methods [124,125], also offer the perspective to obtain relatively pure populations of neurons with functional neuronal features to be used as a differentiation model [118].

Clearly, in vitro neuronal and muscle cells do not present the same level of complexity as a whole organism, and tissue cysts generated in these models have not yet been fully characterized at transcriptomic and metabolomics levels to see if they match better in vivo-derived cysts. However, this provides new interesting perspectives to understand the molecular bases for bradyzoite differentiation and parasite persistence in the host. 

### 5.2. Tools for Genetic Engineering of Mutant Bradyzoites

*T. gondii* is amenable to genetic manipulation and has been used as an important model organism to study general aspects of apicomplexan biology [126]. However, most molecular studies to date have been performed in strains which are relatively easy to grow and to modify genetically, but are poorly cystogenic. 

The ability to manipulate the genome is central to the understanding of *T. gondii* biology and thus for identifying new potential drug targets. Over the years, this has been achieved using several techniques that include genetic crosses [127], random insertional mutagenesis [128,129], chemical mutagenesis coupled with whole genome sequencing [130], genetic editing by homologous recombination [131,132,133], or more recently with the ‘clustered regularly interspaces short palyndromic repeats-Cas9’ (CRISPR/Cas9) system [134,135]. Several of these molecular tools have been used to better understand bradyzoite differentiation. For example, a random insertion-based gene-trapping method was used to identify the *BSR4* gene involved in bradyzoite development [129]. In 2002, Singh and collaborators used chemical mutagenesis coupled with the expression of a reporter GFP under the dependence of a bradyzoite-specific promoter as a selection strategy to isolate mutant parasites unable to convert into bradyzoites [136]. Most importantly, a few years ago, genome editing of a wide variety of organisms was revolutionized by the CRISPR/Cas9-based technology [137]. When implemented in *T. gondii* [134,135], this technique offered the prospect to allow genetic modification of strains otherwise refractory to traditional reverse genetics approaches, including cystogenic strains. Importantly, the CRISPR/Cas9 technique has been used to perform genome-wide screens to predict essential *T. gondii* genes [138,139]. Perhaps the most striking example of the insights this technique can bring into bradyzoite biology, a powerful approach coupling Cas9-mediated screening and single cell profiling has allowed the recent identification of BFD1 as the main transcription factor regulating parasite differentiation [61].

Potential therapeutic targets are often encoded by essential genes whose genetic analysis typically relies on conditional mutants. A number of conditional expression systems can be used in order to tightly control the expression of a gene. Over the last 20 years, several inducible systems enabling to obtain conditional mutants have been successfully applied to *T. gondii* as conditional gene excision with dimerizable Cre-mediated recombination [140], silencing at the transcriptional level with the bacterial tetracycline (tet) operator/regulator system [141], as well as a system for transcript regulation relying on U1 small nuclear ribonucleic particles [142]. Other methods can be used to regulate directly protein levels, using a ligand-controlled mammal-derived FKBP destabilization domain [143], or with the plant-derived auxin-inducible degron (AID) [144,145]. All these strategies depend on the use of small molecules to modulate gene expression. These ligands may be able to cross the cyst wall to efficiently regulate expression in bradyzoites within in vitro-generated cysts, such as with the tet-based system [73]. However, very few of them are predicted to be working efficiently in vivo. In particular, to reach brain-localized in vivo cysts, molecules have to cross the blood–brain barrier, a size-selective filter preventing the passive diffusion of molecules as small as 400 Dalton [146]. Most of these ligands are larger molecules, with the exception of the indole-3-acetic acid (IAA), used to regulate AID. Interestingly, IAA has been shown to successfully control protein degradation in vivo in a cystogenic strain of *T. gondii*, but only in the acute phase of infection [147]. Although its efficiency remains to be investigated during the chronic phase for brain-located cysts, this may be a promising approach. In addition, as there is so far no robust stage-specific inducible system to modulate gene expression in in vivo cysts, strategies that do not involve the use of ligands may be implemented. This could include stage-specific gene excision by expression of a recombinase exclusively at the bradyzoite stage, akin to a strategy developed for *Plasmodium* pre-erythrocytic life cycle stages [148], or down-regulation of a gene of interest upon differentiation by exchanging its native promoter with a tachyzoite-specific promoter.

### 5.3. Emerging Tools for the Phenotypic Characterization of Bradyzoites

The advent of ‘omics’ technologies has enabled the characterization of cell subpopulations at multiple levels including the genome, epigenome, transcriptome, proteome, and metabolome [149]. Several of these techniques require large amounts of starting material, which can be quite challenging when studying bradyzoites. However, some of these approaches improved drastically in sensitivity in the last decade, decreasing the amount of sample required for high-throughput analyses. For instance, datasets covering the global proteome [52,150] or the proteome of specific sub-compartments like the cyst wall [38,39] have been generated from in vitro and in vivo bradyzoites. For the transcriptome, again a series of studies provided datasets describing differential gene expression for in vitro- or in vivo-obtained bradyzoites, using either microarrays [50,117,151], or more recently, the RNA-seq technology [51,52,101]. Metabolomics may be one of the most demanding approaches in terms of amount of starting material and thus has so far remained largely under-investigated in the context of bradyzoites [108]. However, predictive metabolic models can be used to identify metabolic ‘weak spots’ in these developmental stages for further validation by functional analyses [152]. One important challenge is the diversity in the bradyzoite population [34], suggesting potentially different transcriptional [101] or metabolic states within the same cyst. Yet, the emergence of single cell multi-omics technologies now offers the perspective of analyzing minute quantities of molecules and exploring the heterogeneity between cells and within populations previously assumed to be homogeneous [153].

The dynamics of tissue cyst formation and reactivation in the animal model remain largely a black box. Technological limitations have hampered the ability to study even simple aspects of parasite interaction with host tissues. For a long time, imaging of cysts in host tissues was, for example, essentially performed by electron microscopy, a high-resolution but rather time-consuming technique [36]. Recent progress in the genetic engineering of parasites has allowed the generation of fluorescent or bioluminescent reporter cystogenic cell lines, providing an easier way of tracking and imaging of the parasites in the host. The use of bioluminescent parasites, for instance, has been a great tool to assess the formation and the temporal and spatial distribution of cysts in vivo [154]. Progress in fluorescence microscopy techniques also allowed a better monitoring of brain-localized cysts [155,156]. Noticeably, multiphoton imaging now offers the prospect of working on live cells, and has been used to monitor the interactions between *T. gondii* and immune cells in the context of chronic toxoplasmosis [71,157]. In addition, Raman micro-spectroscopy, which can provide chemical and compositional information, has been used to characterize the molecular interactions between *T. gondii* and its host cell microenvironment [158], and may provide interesting insights into the metabolic state of cyst-contained bradyzoites. Overall, recent improvements of both genetic tools and microscopy techniques provide a powerful combination that will likely generate insightful information about host–bradyzoite interaction. 

## 6. Conclusions

Bradyzoites represent a central developmental stage in the pathogenesis caused by *T. gondii*. First, of course, because these slow-growing persistent parasites may reactivate in immunocompromised individuals to give rise to acute toxoplasmosis. But, also because the prospect of about a third of the human population being infected asymptomatically with a brain-dwelling pathogen which is nevertheless potentially capable of causing serious neurological disorders, even in immunocompetent individuals, is not particularly reassuring. Moreover, bradyzoites contained in neuronal cysts are largely resistant to both host immune response and current therapeutics.

New prophylactic or therapeutic strategies are thus direly needed. These should cover not only the human but also the veterinary sides of the disease, as both are intimately linked [159]. For instance, blocking tissue cyst formation or eliminating cysts in food animals, would also contribute to the reduction of this particular source of human infections. Approaches may include drug-based therapeutic solutions to eliminate cysts in contaminated individuals, or a prophylactic vaccine. An attenuated live strain [160], capable of proliferating robustly yet unable to enter a chronic state, may, for instance, be interesting as a vaccine for a veterinary application.

Importantly, identifying drug targets or generating differentiation-deficient strains first necessitates gathering considerable information on basic aspects of the biology of the bradyzoite stage, which has been relatively understudied compared with the tachyzoite stage. There are many outstanding questions that deserve to be investigated in order to better understand key aspects of bradyzoite biology, and this will undoubtedly provide clues and insights for potential future treatments. They include (but are not restricted to): how stress and environmental sensing govern conversion towards latency? How do latent bradyzoites re-enter a proliferative cycle? What are the minimal metabolic requirements for their survival as latent forms? How often do bradyzoites reactivate and how much does it contribute to the persistence of the parasite and the long-lasting immunity of the host? What are the interactions between cyst-contained bradyzoites and brain or immune cells that may lead to behavioral or neurological disorders? Obviously, in spite of recent technical progress, as many of these questions require investigating dynamic processes in the complex and often inaccessible context of the host, they remain difficult to tackle. But hopefully, genetic tools and in vitro models will at least provide interesting insights on potential weak spots that could be exploited for new therapeutic approaches.

Although identifying vulnerable points in bradyzoite biology would provide interesting leads, it may not be sufficient for obtaining efficient therapeutics. Major challenges will have to be met to be able to target these stages with small molecules for instance, like the ability of these drugs to cross the blood–brain barrier [146]. But the main issue may be gaining access to all bradyzoites within large tissue cysts, and effectively targeting heterogeneous populations of parasites that are in different metabolic states. A parallel can be drawn with cancer cells in solid tumors [161] or bacterial persister cells in biofilms [162], which show high variability in accessibility or responsiveness to drugs that contributes to the emergence of resistant lines. However, ultimately, eliminating cysts may be accomplished by: (1) using a drug that is also, or solely, active against bradyzoites; (2) preventing or decreasing altogether the formation of bradyzoites; (3) reactivating dormant bradyzoites into tachyzoites, in order to sensitize them to the lethal action of conventional drugs or of the immune system. Finding the right targets and approaches will necessitate increasing our fundamental understanding of the differentiation and persistence processes, but the renewed interest for chronic toxoplasmosis and the tools now available will certainly lead to future interesting discoveries.

## Figures and Tables

**Table 1:** Factors for inducing in vitro bradyzoite differentiation (IFN, interferon; NO, nitric oxide; IL, interleukine; LPS, lipopolysaccharide; HDAC, histone deacetylase).

Stress Condition	Selected References
*Alkaline pH (pH 8)*	[21]
*Heat shock (43 °C)*	[21]
*Nutrient deprivation*	
Arginine starvation	[18]
Pyrimidine starvation	[26]
Cholesterol deprivation	[22]
*Modulators of immunity and inflammation*	
IFN-Υ	[20]
NO	[16]
IL-6	[25]
LPS	[16]
*Metabolic inhibitors*	
Oligomycin, antimycin A	[16]
Myxothiazol, rotenone, atovaquone	[17]
*Drugs and small compounds*	
Compound 1	[29]
Pyrimethamine	[16]
Sulfadiazine	[27]
Cyclic nucleotide signaling modulators	[28]
Apicidin, FR235222 (HDAC inhibitors)	[19,23,24]
